# Editorial: Kawasaki Disease

**DOI:** 10.3389/fped.2020.631157

**Published:** 2021-01-19

**Authors:** Mamoru Ayusawa, Ichiro Morioka, Adriana Tremoulet

**Affiliations:** ^1^Department of Pediatrics and Child Health, Nihon University School of Medicine, Tokyo, Japan; ^2^Kawasaki Disease Research Center, University of California, San Diego, San Diego, CA, United States

**Keywords:** Kawasaki disease, Tomisaku Kawasaki, vasculitis, infant, coronary artery aneurysm, infant, children

On June 5, 2020, the esteemed Dr. Tomisaku Kawasaki, the pediatrician who first described mucocutaneous lymph node syndrome (later named in his honor) passed away at the age of 95 (1). For 53 years from the time, he published the first 50 cases of this syndrome, he inspired and encouraged researchers around the world to study this elusive illness (2). Since many questions remain regarding Kawasaki disease (KD) in terms of epidemiology, pathology, immunology, cause, and cardiovascular injury, Frontiers in Pediatrics offered us a collection of articles related to KD as a Research Topic. This issue of 13 articles concerning KD in Frontiers in Pediatrics demonstrates the commitment from clinical researchers around the globe to better understand this elusive illness.

This issue includes, for the first time, an explanation of the presentation and outcome of infants with KD and adjunctive therapies for children suffering from KD in a region of the world (Latin America) where little has been previously published about KD. Two comprehensive articles concerning infantile KD cases warn that patients younger than 3–6 months have a higher rate of coronary artery aneurysms. Garrido-Garcia et al., found that these very young infants were particularly at high risk for KD shock and giant coronary artery aneurysms. Moreno et al. found that the admission diagnosis of infants <6 months old ultimately diagnosed with KD was in error in more than 60% of these infants. Fortuna-Reyna et al. detailed for the first time additional therapies administered throughout Latin America for IVIG-resistant KD and aneurysms, revealing a limitation in adjunctive therapies in this part of the world. These works come from the Latin American KD Network (REKAMLATINA- Red de la Enfermedad de Kawasaki en America Latina) (3), inspired by dedicated Latin American physicians that have taken to heart Dr. Kawasaki's call to study this illness. A case report entitled by Saez-de-Ocariz et al. from Mexico highlighted that at times there is a complexity in differentiating KD from other vasculitides.

The work presented also demonstrates a collaborative network of researchers from the US, Europe, and the UK addressing the complexity of the diagnosis of KD with biomarkers, as written in the article by Zandstra et al.. Ching et al. from the Hawaii University investigated a novel biomarker related to cardiovascular dysregulation, and reported the possibility of a novel factor predicting the complication of coronary artery. The risk of hemolysis due to high levels of IVIG therapy, especially in obese children, is addressed in the article by Van Anh et al., which sheds lights on the risk/benefit ratio of IVIG in the context of the obesity epidemic.

Extensive body of work also illustrates the impact of research from Asian countries affected by KD, including Taiwan, China, and Japan. Ming-Huey Guo et al. is an original research article reporting a potentially novel mechanism of macrophage polarization control. The authors found alterations in markers that respond to CpG site methylation in patients with KD. Further, Liu et al. from China, showed swelling of extremities and polymorphous rash, as well as laboratory values, linked to IVIG-resistance. Okubo et al. is a multi-center study from Japan investigated differences in treatment selection and prognosis of the coronary artery outcomes before and after 2012 when the Japanese guidelines shifted toward the utilization of combined immuno-globulin and glucocorticoid therapy. Ito et al. examined the rate of coronary artery aneurysms and IVIG resistance in children administered 30 vs. 50 mg/kg/days during the acute phase of KD. Oshima et al. cautioned about the management of water and electrolytes in the acute phase of severe KD cases. Akimoto et al. discusses the complexities of surgically managing giant aneurysms.

This international effort underscores the importance that we continue to work collectively to understand KD better and honor Dr. Kawasaki's work. We wish to inspire the whole medical community with Dr. Kawasaki's words, “Be Strict for the Medical Study, Be Warm for the Medical Practice.”

## Author Contributions

MA reviewed the articles submitted from Asian countries and edited them, unless he disclosed a conflict of interest as a co-author. IM managed and advised the article collection comprehensively. AT reviewed the submitted articles from Europe and the Americas, and edited them, unless she disclosed a conflict of interest as a co-author. All authors contributed to the article and approved the submitted version.

## Conflict of Interest

The authors declare that the research was conducted in the absence of any commercial or financial relationships that could be construed as a potential conflict of interest.
